# Compact lightweight imager of both gamma rays and neutrons based on a pixelated stilbene scintillator coupled to a silicon photomultiplier array

**DOI:** 10.1038/s41598-021-83530-4

**Published:** 2021-02-15

**Authors:** Jihwan Boo, Mark D. Hammig, Manhee Jeong

**Affiliations:** 1 Nuclear and Energy Engineering, Jeju National University, Jeju, 63243 Republic of Korea; 2 Nuclear Engineering & Rad. Sci., University of Michigan, Ann Arbor, 48109 USA

**Keywords:** Energy science and technology, Engineering

## Abstract

Dual particle imaging, in which both neutrons and gamma-rays in the environment can be individually characterized, is particularly attractive for monitoring mixed radiation emitters such as special nuclear materials (SNM). Effective SNM localization and detection benefits from high instrument sensitivity so that real-time imaging or imaging with a limited number of acquired events is enabled. For portable applications, one also desires a dual particle imager (DPI) that is readily deployable. We have developed a hand-held type DPI equipped with a pixelated stilbene-silicon photomultiplier (SiPM) array module and low sampling-rate analog-to-digital converters (ADCs) processed via a multiplexed readout. The stilbene-SiPM array (12 × 12 pixels) is capable of effectively performing pulse shape discrimination (PSD) between gamma-ray and neutron events and neutron/gamma-ray source localization on the imaging plane, as demonstrated with ^252^Cf neutron/gamma and ^137^Cs gamma-ray sources. The low sampling rate ADCs connected to the stilbene-SiPM array module result in a compact instrument with high sensitivity that provides a gamma-ray image of a ^137^Cs source, producing 6.4 μR/h at 1 m, in less than 69 s. A neutron image for a 3.5 × 10^5^ n/s ^252^Cf source can also be obtained in less than 6 min at 1 m from the center of the system. The instrument images successfully with field of view of 50° and provides angular resolution of 6.8°.

## Introduction

The nuclear safeguarding of special nuclear materials (SNM), such as plutonium and highly-enriched uranium, requires instruments that can detect, localize, and quantify the isotopic content of the target^1^. The accuracy of the material assessment can be enhanced if both the neutron and gamma-ray emissions are interrogated by a dual-particle imager (DPI). When coupled with a neutron-interrogating source, a DPI based on electronic collimation can localize a highly enriched uranium (HEU) solid surrounded by neutron shielding and distinguish it from a dummy material item such as tungsten^2^. The DPI system described in^3^, which utilizes a coded aperture, successfully images both ^241^AmBe and ^252^Cf photon/neutron sources.

Despite these demonstrations, DPI systems have not yet been designed to be more portable and deployable or to maintain sufficiently high sensitivity to rapidly reconstruct images. The aforementioned DPI using electronic collimation employed a large, bulky single crystal array of EJ-309 and NaI:Tl in which each scintillator was coupled to a photomultiplier tubes (PMT), and each PMT was processed by its own devoted readout circuit. Furthermore, the channel readout was performed with cumbersome digitizers such as the CAEN V1720, which cannot be applied to hand-held use due to its size. A previous study on this DPI system also quantified the low efficiency (10^−4^ per incident neutron or photon) with which it created imageable events^4^. On the other hand, the DPI system in^3^ based on the coded aperture has an array of bulky single crystals whose size are inversely proportional to the angular resolution of the system. In addition to its size, it took an hour each to measure the sources using mask and anti-mask measurement configurations, an approach that prevents real-time imaging.

In^5^, the authors implemented a pixelated detector in a hand-held DPI configuration using pulse-shape discrimination (PSD). The instrument, which consisted of a plastic scintillator (EJ-299-34) 13 × 13 square array with a small pixel size (2.8 × 2.8 × 15 mm^3^), exhibited a marked degradation of PSD performance in the pixelated crystal array when compared to a single crystal. The PSD performance of the pixelated array, however, could not yet be fully verified because the authors detected the ^252^Cf stored in a water tank that reduced the average energy (2.1–2.5 MeV) of the fast neutron by one-third (to 0.7–0.9 MeV). Furthermore, because the estimation of PSD performance was performed across the entire energy spectrum, the results were biased toward the low energy events that generally represent the highest misclassification probability in most organic scintillators^6^. They were also not capable of showing pixel-by-pixel PSD performance because the pixelated array was mounted on a single-channel photomultiplier tube (PMT) rather than pixelated photodetectors, such as silicon photomultiplier (SiPM).

There have been recent efforts to develop a hand-held DPI system^7^ using high sampling rate analog-to-digital converters (ADCs), in which stilbene scintillator bars were coupled to an SiPM. Stilbene was chosen because it has high light output and high PSD performance when compared to other organic scintillators^8^. The SiPM also provides a small footprint and low voltage/power operation compared with PMTs. Nevertheless, the hand-held DPI system required high sampling rate ADCs (e.g., ADCs with 14 bits and 500 MS/s) in order to provide sufficient time resolution to identify coincident interactions for time-of-flight (TOF) implementations. High-performance ADCs pose a limit in the use of portable DPI systems due to their high power consumption and large footprint. Because each stilbene bar is combined with two SiPM arrays that were both individually readout^9^, increasing the number of these bars in future studies will double the number of readout channels and therefore increase the number of ADCs. An additional cooling system may be required for the ADCs unless a multiplexing method is implemented.

Another important performance consideration for many deployed or mobile applications is ensuring that the sensitivity of the DPI system is high enough to rapidly reconstruct images. As the SNM sample-to-detector distance increases, the validity of the SNM detection can rapidly decrease^10^. The aforementioned hand-held DPI system requires a long measurement time to reconstruct a neutron image of SNM as described in^7^ due to its small number of stilbene bars. Furthermore, the complexity of the data collection system, such as determining the TOF between neutron interactions can increase dead-time losses and restrict the efficiency of the system. For instance, the minimum time difference between coincident neutron events should be 250 ps or more and the energy of the neutron after the initial scatter (E_TOF_) should be greater than the energy deposition in the second interaction. Thus, the minimum energy neutron required to generate an imageable event is roughly 1.5 MeV. The applied correction factor also created image artifacts in the reconstructed gamma-ray image because the entire energy of the gamma-ray is not fully deposited in the stilbene scintillators.

In this paper, we demonstrate the performance of a coded-aperture based hand-held DPI system using a pixelated stilbene-SiPM array module and low sampling rate ADCs. To our knowledge, this also represents the first experimental results of PSD performance when using a pixelated stilbene scintillator coupled to a SiPM array implemented with a multiplexing method such as row/column summing readout. The stilbene-SiPM array (12 × 12 pixels) module has high sensitivity that limits the measurement time for gamma-ray and neutron image acquisition. The coded-aperture based system utilizes the low sampling-rate ADCs to extract the relevant pulse information derived from neutron and gamma events in the detector module, while keeping the instrument mass of 4.1 kg and the device size compact.

## Results and discussion

**Figure 1:**
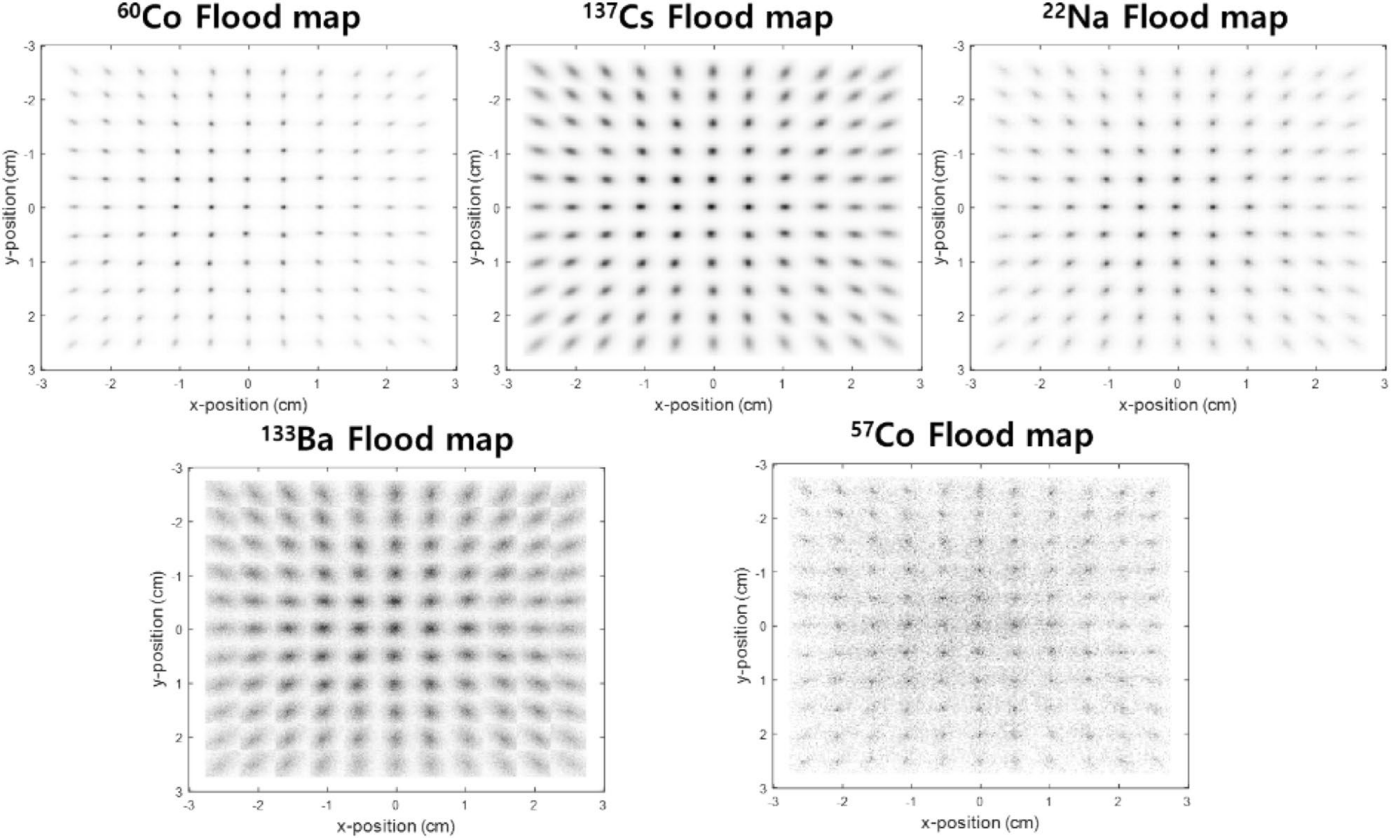
Measured 2D flood map of 12 × 12 pixels in stilbene-SiPM array module for ^60^Co, ^137^Cs, ^22^Na, ^133^Ba, and ^57^Co gamma irradiation. The source activity of each gamma-ray source was 0.312 MBq and the 10 cm distant source was measured for 1 h. The 12 × 12 stilbene scintillator had pixel dimensions 4 × 4 × 20 mm^3^.

**Figure 2:**
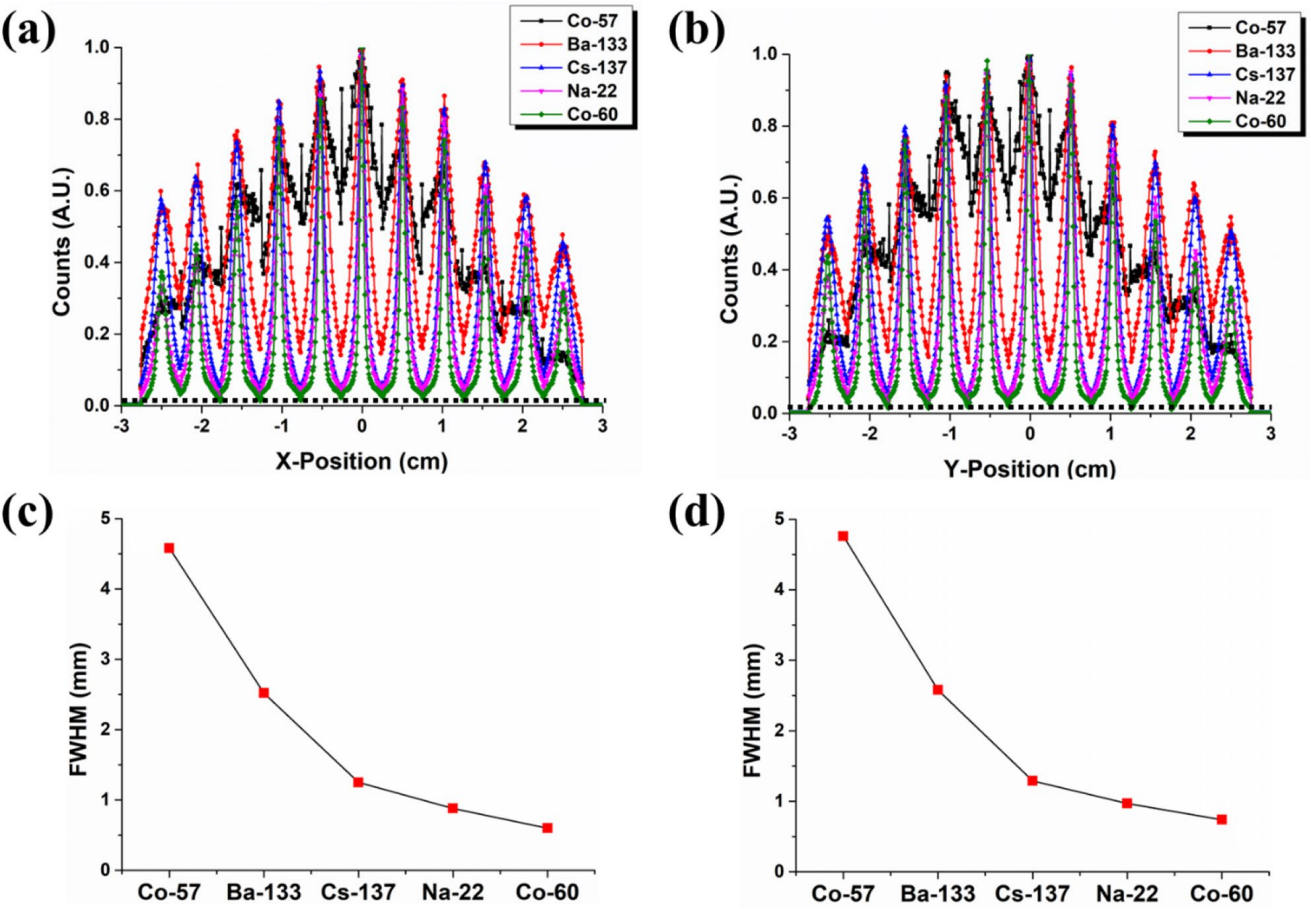
(**a**) 1D row-sum profile and (**b**) 1D column-sum profile derived from the flood map in Fig. 1 The corresponding FWHM resolution of the pixel-center for all row pixels (**c**) and column pixels (**d**) as the radionuclide is varied.

Figure 1 shows measured 2D flood maps that all 144 elements of a stilbene-SiPM array module can be identified when exposed to either ^60^Co, ^137^Cs, ^22^Na, ^133^Ba, or ^57^Co gamma-ray sources (0.312 MBq activity) located 10 cm from the face of the module for an hour. The corresponding 1D slices through the center pixels of the detector map are shown in Fig. 2 for various gamma-ray sources. As the gamma-ray energy decreases, the detection efficiency increases as expected; however, Fig. 2 shows that the positional resolution across the array can degrade as the energy deposited is diminished. Note in particular that the relatively small 122 keV and 136 keV energy depositions from ^57^Co result in significantly poorer localization in part because the signal size is degraded by competing electronic noise. In particular, the pixel-center is identified for all row or column pixels with a FWHM resolution that varies from 0.6 mm for the higher energy emitters to 4.76 mm for ^57^Co.

As shown in both Figs. 1 and 2, the positional degradation for lower-energy depositions reflects a higher relative participation of multi-pixel scattering events due to more intense sampling of large Compton scattering-angle events. Moreover, if there is optical cross-talk between pixels due to the imperfect capture of scintillation photons from the interaction pixel, then positional blurring and a mislocation of the interaction location can result. This occurs because the SiPM pixel area and the scintillation pixel area do not precisely match. One can align the scintillating light sources with the individual photosensors across the array due to the fact that the scintillator pixel pitch (4.2 mm) is identical to the SiPM. Nonetheless, each scintillator pixel with a size of 4 × 4 mm^2^ overlays a SiPM readout pixel with only a size of 3 × 3 mm^2^, causing a dead space. Thus, the SiPM dead space can potentially allow optical cross-talk. These negative impacts could be alleviated when considering only those events that occur close to the center of the pixel. The multiplexing scheme used here could set the acceptance range specified by the user as only those events that deposit their energy within 20% of the distance between the two pixels. This could be realized by the fact that the end of each row/column summing readout provided the signals that identified all interaction positions in real-time. This event processing manner could also give a positive effect on neutron measurement as we can effectively minimize multi-pixel events.

**Figure 3:**
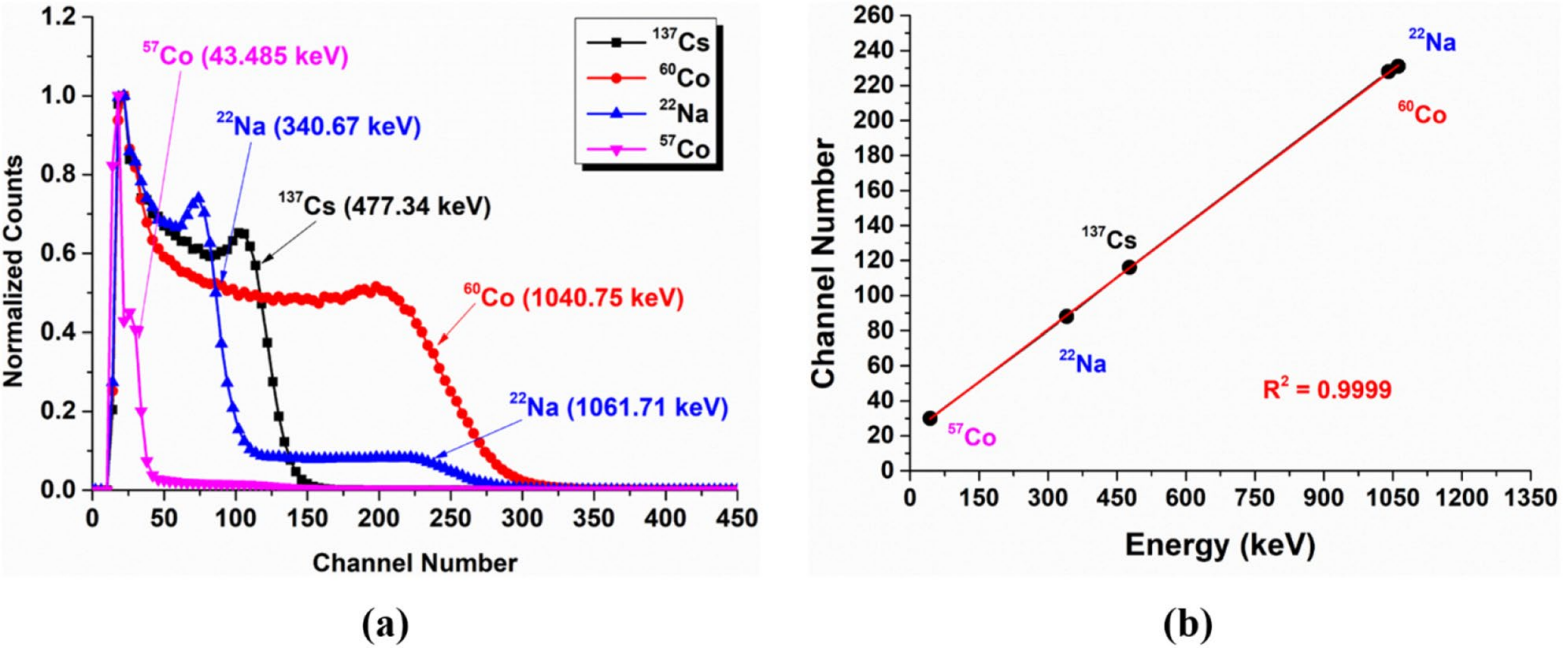
(**a**) Energy spectra of the stilbene scintillator array coupled to the SiPM array when irradiated by the gamma-ray sources shown. (**b**) Measured Compton edge position (in channel number) as a function of analytical Compton edge energy when the SiPM was operated at a 28 V bias voltage and a temperature of 28℃. The Compton edge position was calculated at the channel number corresponding to 70% of the Compton peak for each of the gamma-ray sources.

As shown in Fig. 3, energy spectra were acquired using the gamma-ray sources, and an energy calibration to units of keVee was made that had high accuracy (R^2^ > 0.9999) in the range of Compton edge energy depositions between 40.385 and 1061.71 keV. The positional determination of the Compton edge was taken as that channel number that corresponded to the distribution position that was 70% of the Compton peak for each of the gamma-ray sources^11^. We confirmed that energy linearity was maintained after the electronic gain adjustment as expected because although there is pixel-by-pixel light yield and gain factor variation, those differences are compensated for by pixel-by-pixel gain calibrations for every element coupled to the SiPM^12^. An energy calibration to units of keVee was subsequently made using the linear light output response functions to photon interaction.

**Figure 4:**
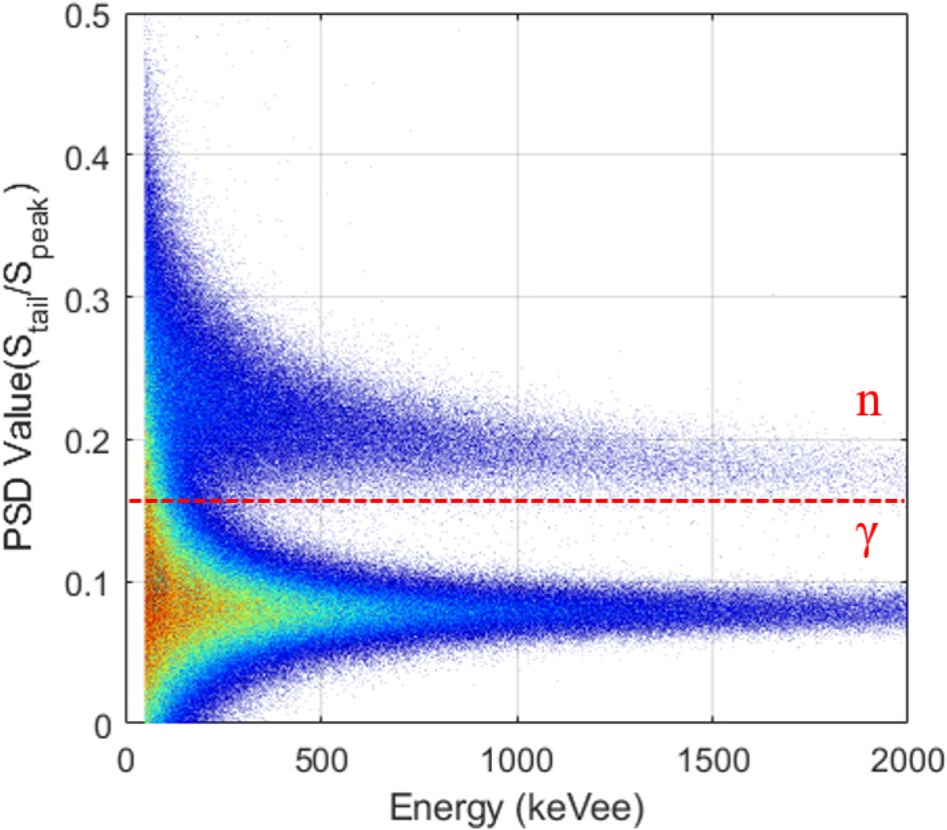
*PSD* plot for 1,800,000 pulses with an energy threshold of 50 keVee produced by the stilbene-SiPM array module measuring a 3.5 × 10^5^ n/s ^252^Cf spontaneous fission source located 50 cm away from the front of detector. The dashed *PSD* cutoff line is established by the user.

**Figure 5:**
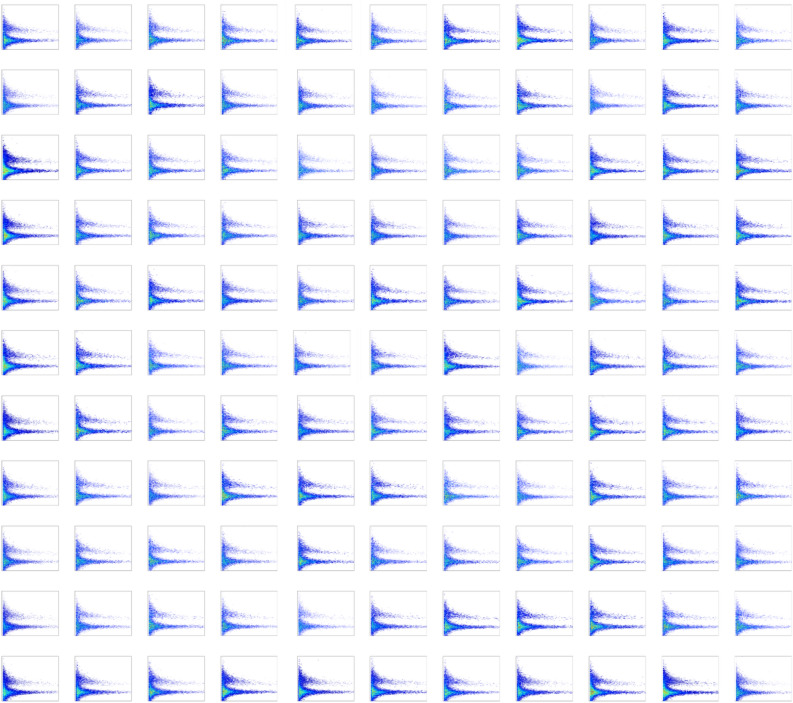
Pixel-by-pixel *PSD* plot for each 4 × 4 × 20 mm^3^ stilbene scintillator pixel from which the *overall* detector *PSD* distribution in Fig. 4 is generated.

The pulse shape discrimination metric plot is shown in Fig. 4, which was acquired by measuring a 3.5 × 10^5^ n/s ^252^Cf spontaneous fission source 50 cm distant from the detector module. The description and evaluation of the PSD metric is detailed in the Methods section. Gamma-ray events are dominant below the dashed line shown in the figure while the neutron events are predominant above the dashed line. At the energy of 200 keVee, the *overall* detector *PSD* plot has a partial overlap between the gamma-ray and neutron distributions that makes it difficult to isolate the relevant particle species; however, once *PSD* sorting is applied for each pixel, as shown in Fig. 5, a clearer distinction between the neutron and gamma-ray events above the energy of 100 keVee is observed. The measurements thus indicate that gamma/neutron discrimination is achievable on a pixel-by-pixel basis.

**Figure 6:**
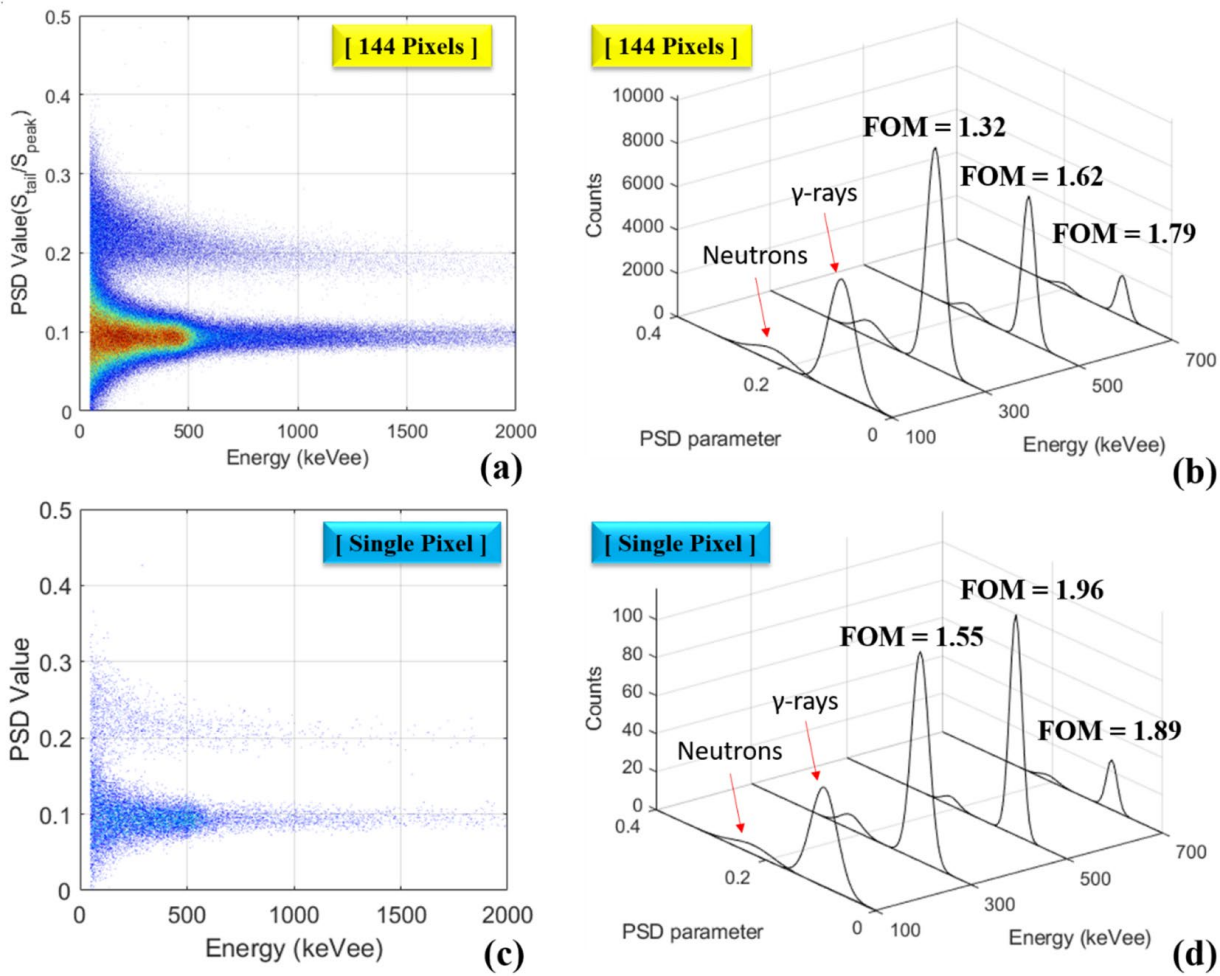
(**a**) The *overall* detector *PSD* plot for 1,500,000 pulses produced by the stilbene-SiPM module with a threshold of 50 keVee by measuring a 3.5 × 10^5^ n/s ^252^Cf spontaneous fission source at 75 cm distance and a ^137^Cs source producing 30.16 μR/h at 30 cm distance. (**b**) The distribution of *PSD* parameter at various energies (300 ± 100 keVee, 500 ± 100 keVee, 700 ± 100 keVee) for the *overall* detector *PSD* plot. (**c**) The *PSD* plot that one of the pixels has and (**d**) the corresponding FOM values for the aforementioned three cases.

In addition to the *PSD* plot in Fig. 6a, a figure of merit (*FOM*) that measures the effectiveness of the gamma/neutron discrimination is defined in the Methods section and shown graphically in Fig. 6b. The FOM profiles that visualize the neutron/gamma-ray separation at different energy regions: 300 ± 100 keVee, 500 ± 100 keVee, 700 ± 100 keVee, are presented, when the stilbene detector is exposed to combined ^252^Cf and ^137^Cs sources. The neutron/gamma ^252^Cf source was located 75 cm distant and the ^137^Cs gamma-ray source producing 30.16 μR/h was located 30 cm away from the detector module. The high intensity of counts in the 300 to 500 keVee region of the gamma-ray section of the *PSD* plot correspond to the Compton edge and the Compton continuum regions of ^137^Cs in Fig. 6a. This region buttresses the reliability of the PSD method and its relationship with energy correction to units of keVee. Figure 6c shows the *PSD* plot that one of the pixels has, and the corresponding *FOM* values higher than 1.55 were achieved for the aforementioned three cases, as shown in Fig. 6d. It should be noted that a single stilbene scintillator block coupled to the PMT shows better neutron/gamma separation performance when using digitizers with a sampling frequency of 500 MHz and 14-bit resolution^13,14^. The values of the *FOM* quoted in these studies (∼1.94–2.5) are considerably higher than those shown in the current study. This is because the stilbene scintillator pixels used here are not only smaller than those used in the reference studies, but the stilbene scintillator used here has a smaller pixel size than the active area of SiPM pixel. Thus, the light loss might cause a lower *FOM* value. Nevertheless, because intrinsic particle separation of the scintillator is achieved^5^, the low sampling rate ADCs were capable of performing satisfactory PSD using the pixelated stilbene-SiPM array module.

**Figure 7:**
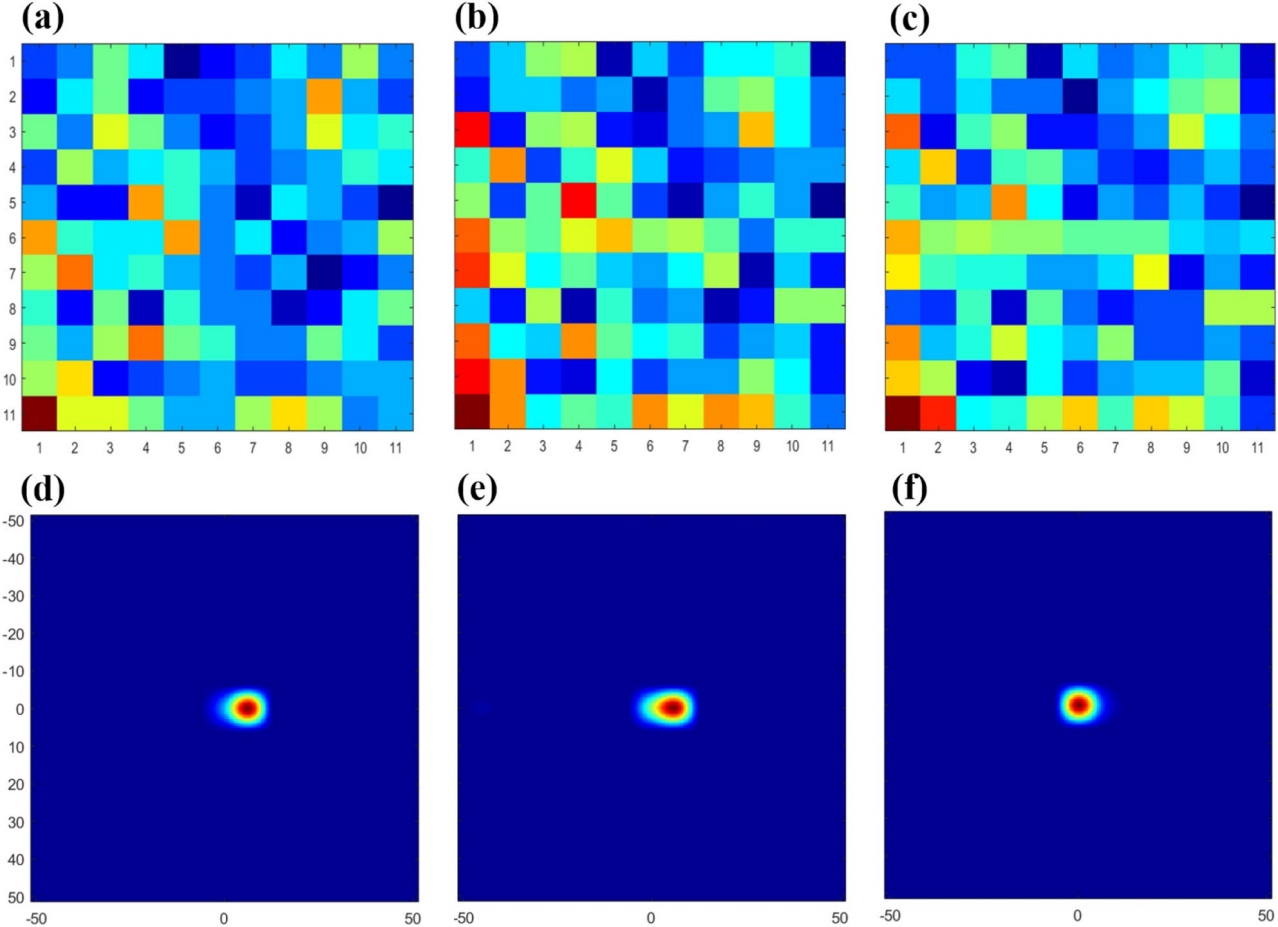
Comparison of reconstructed images of neutron when exposed to a 3.5 × 10^5^ n/s ^252^Cf spontaneous fission source at 100 cm distance. Detector map composes of the neutron events obtained for 1 min (330 events) (**a**), 3 min (1,000 events) (**b**), and 6 min (2,000 events) (**c**). The three detector maps correspond to the images reconstructed by using MLEM (**d–f**), respectively.

Sensitivity tests were conducted to study the reconstructed image quality and localization accuracy by increasing either the measurement time or the number of recorded events, as shown in Fig. 7. The ^252^Cf spontaneous fission source was measured at 100 cm from the center of the system, using the hand-held DPI system equipped with a centered-mosaic modified-uniform redundant array (MURA) mask detailed in the Methods section. The collected data were binned in an energy window^15^ with a minimum threshold of 50 keVee. For quantitative and qualitative evaluation on the images, the reconstructed images were compared with reference ground-truth images (i.e. spatial distributions filled with 0 except for a 1 at the image center) through peak signal-to-noise ratio (*PSNR*)^16^, normalized mean-square error (*NMSE*)^17^, and structural similarity (*SSIM*) metrics^18^. If the *PSNR* value exceeds 33 dB or the *NMSE* value is close to 0, or the *SSIM* value is close to 1, it is judged that the reconstructed image is hard to distinguish from the original object.

Figure 7a-c show detector maps consisting of 330, 1,000, and 2,000 neutron events, respectively, classified as higher than the *PSD* value of 0.16 following the binning of the data. The corresponding MLEM images were reconstructed using each of these detector maps, as shown in Fig. 7d-f. These three images correspond to measurement of 1 min, 3 min, and 6 min, respectively (a figure verified using repeated experiments). Table 1 presents the results of the neutron image evaluation using the three imaging metrics (*PSNR, NMSE, SSIM*) mentioned above. As the number of neutron interactions increased from 1,000 to 2,000, the *PSNR* metric surpassed 33 dB- and in fact, increase to 39.29 dB, the *NMSE* decreased by more than an order of magnitude to ~ 10^−4^, and *SSIM* approached closely to 1, the combined evaluation of which indicated that effective localization was achieved and imaging artifacts were minimized. The experiments verified that the coded aperture-based DPI can provide effective neutron-source imaging with a limited number of counts and therefore rapid imaging for relatively intense or proximate neutron sources. This can be compared with H2DPI from the University of Michigan^5^ that has a quoted localization time of 30 min for the neutron image of a 1.2 × 10^7^ n/s ^252^Cf source at 58.4 cm from the center of system.

**Table 1:** Neutron image quality evaluated by using PSNR, NMSE, SSIM as increasing measurement time or the number of counts.

Number of neutron events above 200 keVee	PSNR	NMSE	SSIM
330 (1 min)	22.90	4.7 × 10^−3^	0.81
1,000 (3 min)	23.74	3.3 × 10^−3^	0.85
2,000 (6 min)	39.29	1.1 × 10^−4^	0.99

**Figure 8:**
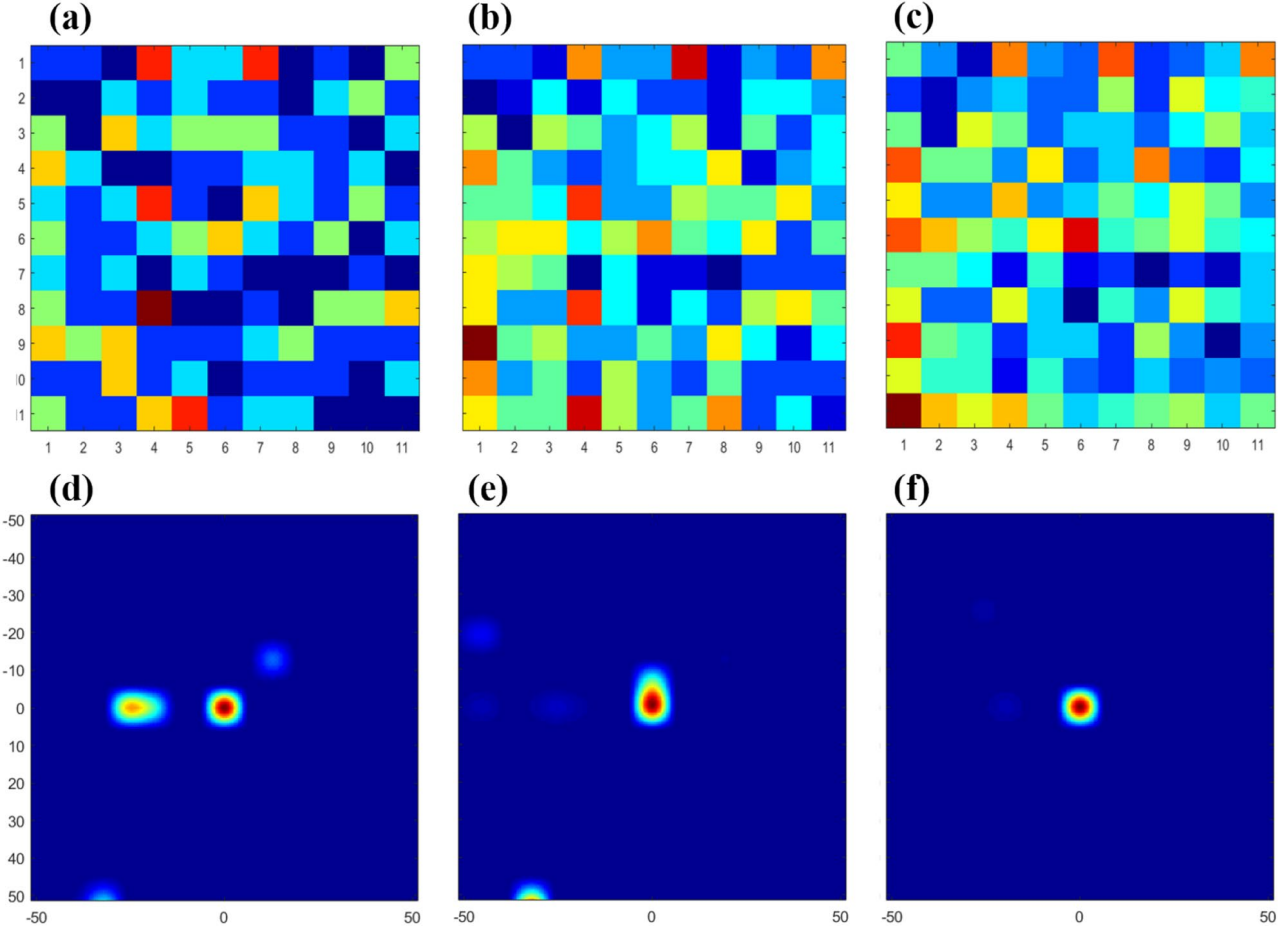
Comparison of reconstructed images of gamma-ray events when exposed to a ^137^Cs source producing 6.4 μR/h at 100 cm distance. The detector map composed of the gamma-ray events for (**a**) 20 s (300 events), (**b**) 40 s (600 events), and (**c**) 69 s (1,000 events). The three detector maps correspond to the images reconstructed by using MLEM (**d–f**), respectively.

Similar to neutron image evaluation, we estimated the quality of gamma-ray images by measuring the ^137^Cs producing 6.4 μR/h at 100 cm from the hand-held DPI. Following the same process of binning the data, Fig. 8a-c presents each detector map consisting of 300, 600, and 1,000 gamma-ray events which are lower than the PSD value 0.16, and MLEM images can be obtained using these detector maps, respectively. The three images in Fig. 8d-f correspond to measurement times of 20 s, 40 s, and 69 s, respectively, figure qualities verified via repeated measurements. The results of gamma-ray image evaluation using the three factors are presented in Table 2. As the classified count number surpasses 1,000, the high *PSNR* (50.65 dB), low *NMSE* (~ 10^−5^) and near-unity *SSIM* buttresses the qualitative evaluation of the Fig. 8 images which indicate that the gamma-ray source is effectively localized with minimal imaging artifacts. A previous study^19^ in our laboratory supports this image-quality result because the number of approximately 1,000 counts per second provided real-time image reconstruction when using the hand-held coded aperture gamma-ray imaging system with a pixelated 12 × 12 *inorganic* scintillator-SiPM array module.

**Table 2:** Gamma-ray image quality evaluated by using PSNR, NMSE, SSIM as increasing measurement time or the number of counts.

Number of gamma-ray events above 200 keVee	PSNR	NMSE	SSIM
300 (20 s)	25.17	1.6 × 10^−3^	0.73
600 (40 s)	26.82	1.3 × 10^−3^	0.82
1,000 (69 s)	50.65	8.6 × 10^−6^	0.99

**Figure 9:**
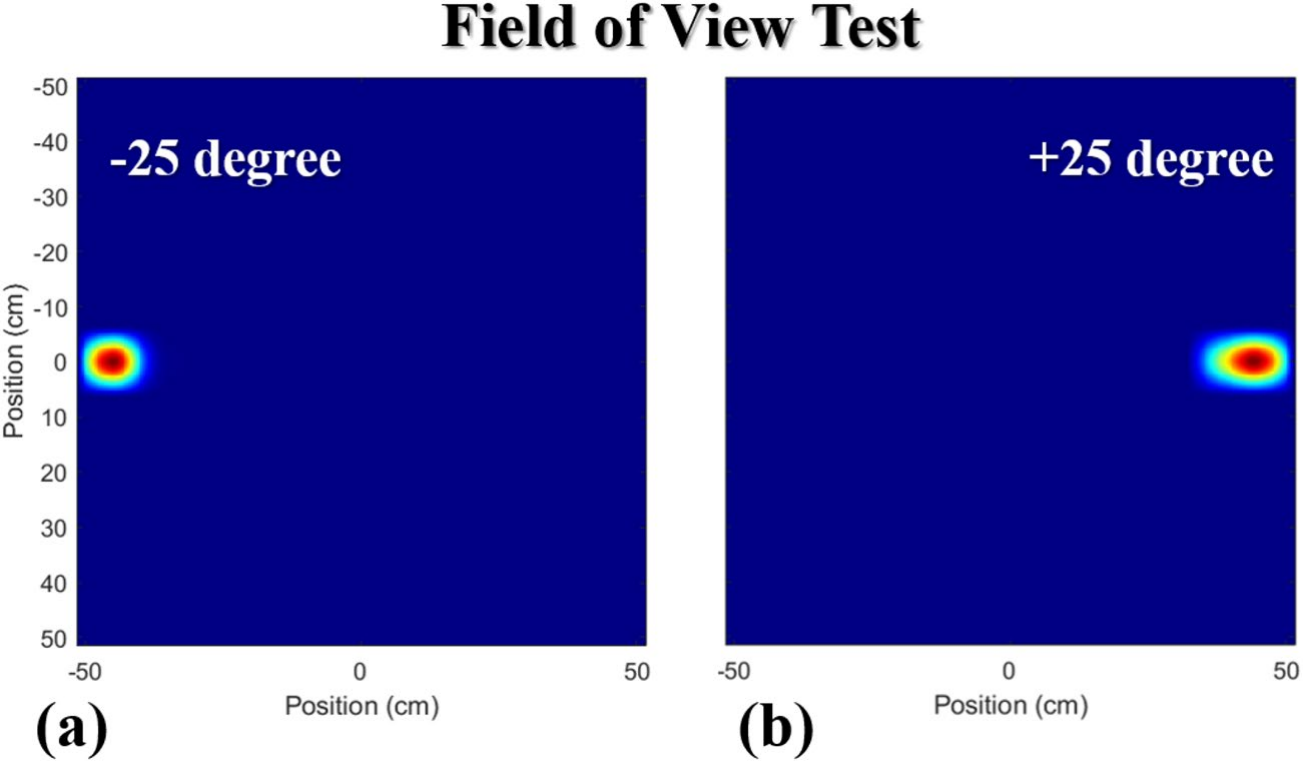
Field of view (*FOV*) tests for the coded-aperture DPI system when a ^137^Cs source of 0.312 MBq located at an angle of − 25° (**a**), and at + 25° (**b**) at 100 cm distance. Both reconstructed images are obtained using the gamma-ray events above the 50 keVee.

Figure 9 shows the test results that evaluate the maximum angular field of view (*FOV*) of the hand-held coded aperture based DPI system. The ^137^Cs gamma-ray source position was varied over angles from − 25° to + 25°, located 100 cm away from the imaging device. The source position is well-reconstructed across the angular range. Note that the elongation in the + 25° image compared to the − 25° image is due to the count sharing between neighboring pixels on the coarsely sampled image plane. Moreover, compared with the ~ 1,000 counts required to form high-quality images for sources near the center of the *FOV*, an average number of 1,800 events was required to obtain acceptable accuracy of the source position and image quality. The increased measurement time was needed to account for the increased participation of gamma-rays that were scattered by the mask itself at the higher incident angles. This degradation mechanism can be mitigated by using energy windowing techniques that sort out only the Compton edge events of ^137^Cs^20^.

**Figure 10:**
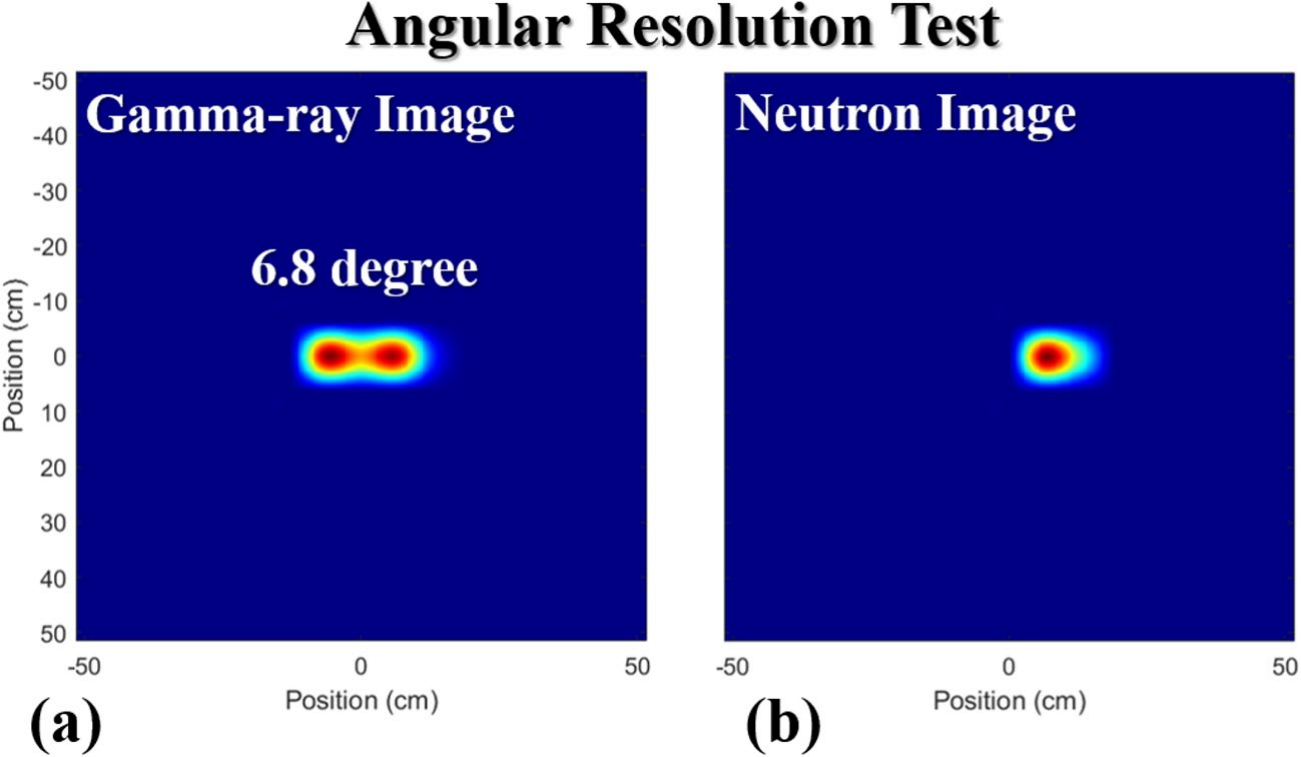
Angular separation test for the coded-aperture DPI system when exposed to a 3.5 × 10^5^ n/s ^252^Cf spontaneous fission source at 75 cm distance and a ^137^Cs source producing 30.16 μR/h at 30 cm distance separated by 6.8°. Reconstructed images using gamma-ray events measured for 2 min (**a**) and for neutron events for 4 min (**b**) over the energy range above 50 keVee.

For the angular resolution demonstration, we previously established that the angular resolution derived from a GAGG:Ce gamma-ray imaging system was 6.8°^19^. The 3.5 × 10^5^ n/s ^252^Cf spontaneous fission source at 75 cm distance and a ^137^Cs source producing 2.71 μR/h at 30 cm were thus separated by an angle of 6.8°. When utilizing the detector map obtained from gamma-ray events that are in the energy range above 50 keVee and have the *PSD* value less than 0.16, the two sources were distinguished in the gamma-ray image using MLEM as shown in Fig. 10a. It should be noted that on average 7,000 gamma-ray events (2-min measurement time), through repetitive experiments, ensured the successful separation of two sources in the image. The neutron image in Fig. 10b shows a hot-spot in the correct ^252^Cf position and no source in the ^137^Cs location. An average of 2,000 neutron events (4-min measurement time), are required to retain the image quality and localization accuracy. A higher overall count was required to obtain these source images because the signal-to-noise ratio decreases as multiple sources are exposed to the coded aperture imager^21^.

**Figure 11:**
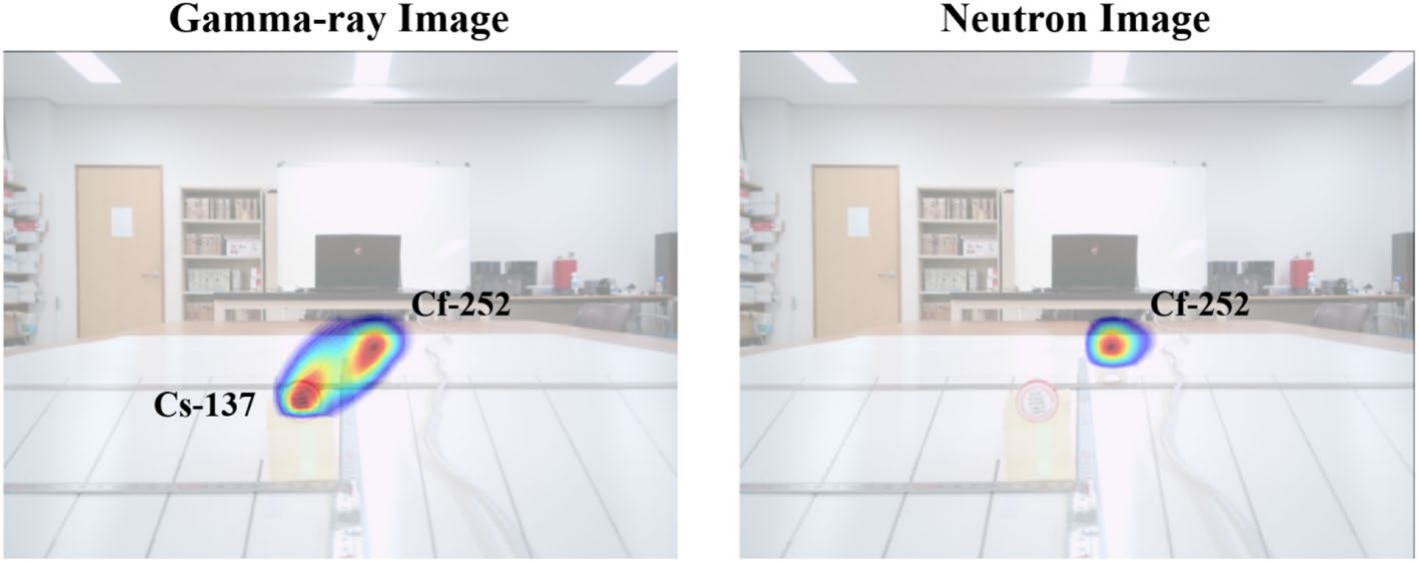
The optical camera images superimposed with the reconstructed images shown in Fig. 10.

Figure 11 shows the radiographic images superimposed upon an optical image generated by a complementary metal oxide-semiconductor (CMOS) image sensor (IMX214 produced by SONY). In image superimposition processing, homography estimation^22^ was used to convert a distorted plane viewed from the front into a two-dimensional image plane that has a perspective projection. Similar to performing a perspective transformation, matching between two images can be performed through the coordinate shift information of nine corresponding points, and the homography matrix can be obtained from matching information using the random sample consensus (RANSAC) algorithm^23^. When using the calculated homography matrix we were able to reconstruct the scene and infer the presence and location of mixed gamma-ray and neutron point sources.

## Conclusion

In this paper, a hand-held coded aperture based DPI system was realized by processing the signals from a 12 × 12 pixelated stilbene-SiPM array module via a row/column summing readout. The coded aperture based system maintained a weight of 4.1 kg and compact size by utilizing low sampling-rate ADCs (50 MHz) that extract the relevant pulse information derived from neutron and gamma-ray events in the detector module. The system shows excellent energy linearity, good neutron/gamma discrimination ability, intrinsic detection efficiency, and imaging quality when measuring various gamma-ray sources and a ^252^Cf spontaneous fission source. The low sampling rate ADCs connected to the stilbene-SiPM array module are capable of performing satisfactory PSD from the stilbene scintillator. In addition, future plans include the upgrade of the ADCs that have 14-bit resolution to improve PSD performance^24^ while retaining the present sampling rate of 50 MHz. Higher ADC resolution improves the accuracy with which one faithfully represents the analog signal shape, which can allow one to discriminate gamma-rays and neutrons with more confidence. In particular, reducing the degree of overlap in the neutron and gamma-ray PSD signals under an energy of 100 keVee lowers the measurement time that is required to accurately classify neutron and gamma-ray events and form radiation images. We also expect, but have not yet proven, that one can minimize optical cross-talk between pixels- if it exists due to imperfect matching between scintillator and SiPM pixel sizes as in our case- as well as multi-pixel events by considering only those events that occur close to the center of the pixel because the row/column summing readout identified all interaction positions in real-time.

## Methods section

### Hardware fabrication and configuration

**Figure 12:**
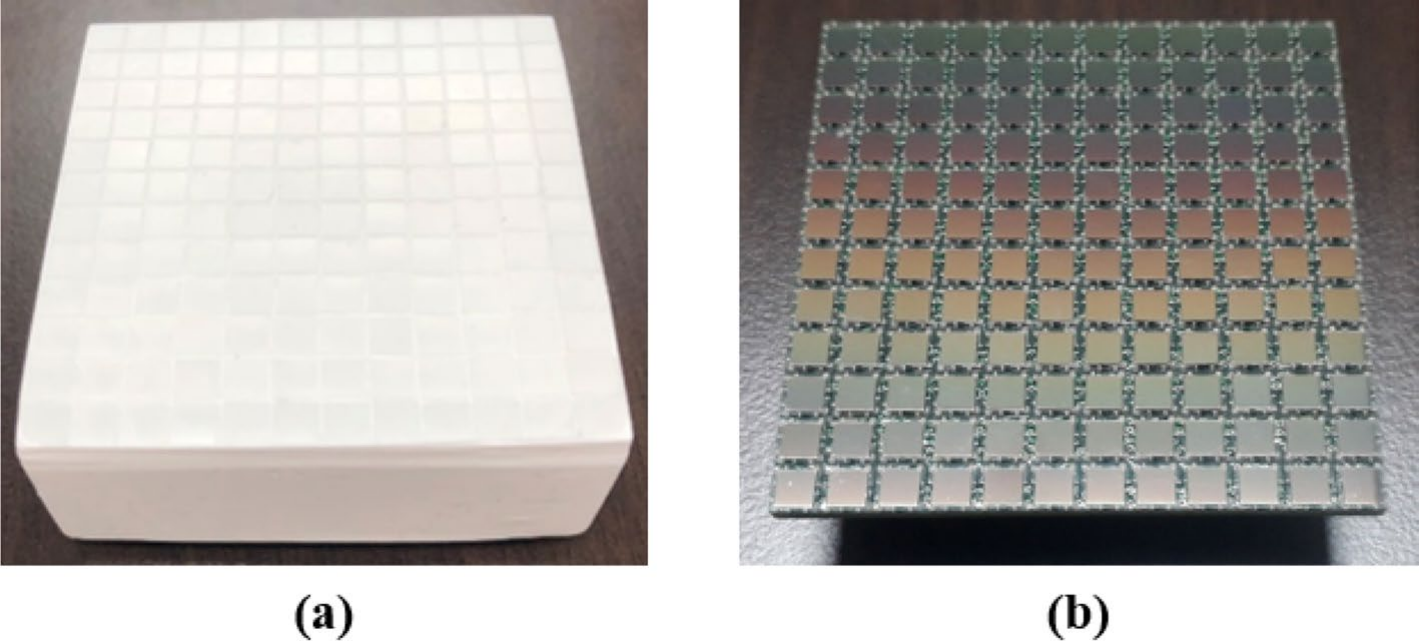
Stilbene scintillator array (12 × 12 pixels of 4 × 4 × 20 mm^3^ each) (**a**), and SiPM array with the same number and area of pixels (**b**).

**Figure 13:**
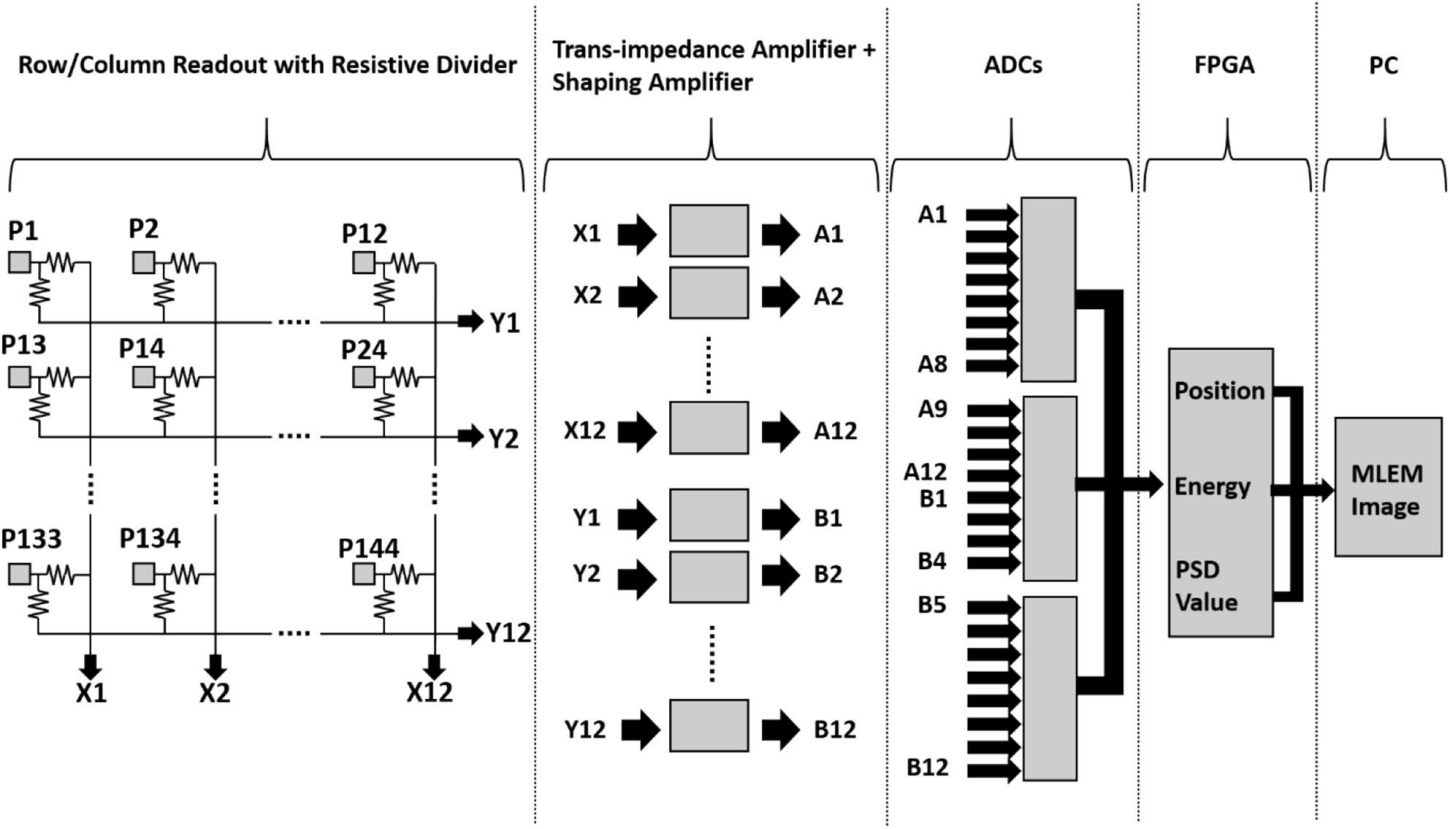
Hardware configuration with row/column summing readout and 3 ADCs for 144 pixels upon the SiPM array coupled to stilbene scintillator array implemented on coded-aperture hand-held imaging system.

This hand-held DPI system is developed for neutron/gamma detection by using the 12 × 12 stilbene scintillator array (produced by Inrad Optics) which has a pixel size of 4 × 4 × 20 mm^3^ and bound by 0.1 mm of polytetrafluoroethylene (PTFE) reflective material, resulting in a 4.2 mm pixel pitch and a 50.2 × 50.2 mm^2^ active area, as shown in Fig. 12. The stilbene scintillator array, directly coupled with the SiPM array (ArrayC-30035-144P) with the same number and area of pixels, was covered with 3 layers of Teflon tape. Note that no index-matching coupling compound was used between the scintillator and the photosensor as we found that the increased distance from the scintillator to the photosensor that accompanied its application worsened the imaging resolution more than the improvement accrued from any increased photon transmission. The SiPM array sensor has both fast and standard outputs and only standard outputs are used in this work. The readout scheme, implemented with the circuit shown schematically in Fig. 13 makes use of a row/column summing readout with a resistive divider^25^. The readout circuit reduces the standard outputs from 144 to 12 for each orthogonal X and Y direction in which the interaction position of individual scintillation events is determined. The X and Y line outputs are connected to 24 transimpedance preamplifiers each having a transimpedance of 49.9 Ω, followed by individual shaping amplifiers. The feedback resistance was chosen as that which produced a sufficiently low noise level at the preamplifier (larger resistances producing higher noise) while also minimizing pulse shape distortion, the degree of which decreases as the resistance decreases.

The amplified analog signals from columns and rows were digitized individually by three low-sampling rate ADCs (50 MHz, 8 channel, 12-bit, ADS5281, Texas Instruments). The energy of the event was calculated as the moving sum of all ADC values (digitized every 20 ns) derived from the columns and rows within a predetermined time window of 640 ns set by firmware. When the summed value is larger than a given threshold set in the field programmable gate arrays (FPGA, XC7K70T-1FBG484C, Xilinx) by the user, a trigger signal is generated to take data. If the summed ADC value exceeds the trigger level, the event position is also determined for the pixel that has the largest signal in its column and row. This method is less sensitive to noise than Anger logic because noise is not summed for as many channels. When two positions are different from each other by more than the given threshold, the trigger is canceled and the event is discarded (we consider this event as a multi-pixel event inside crystal array).

**Figure 14:**
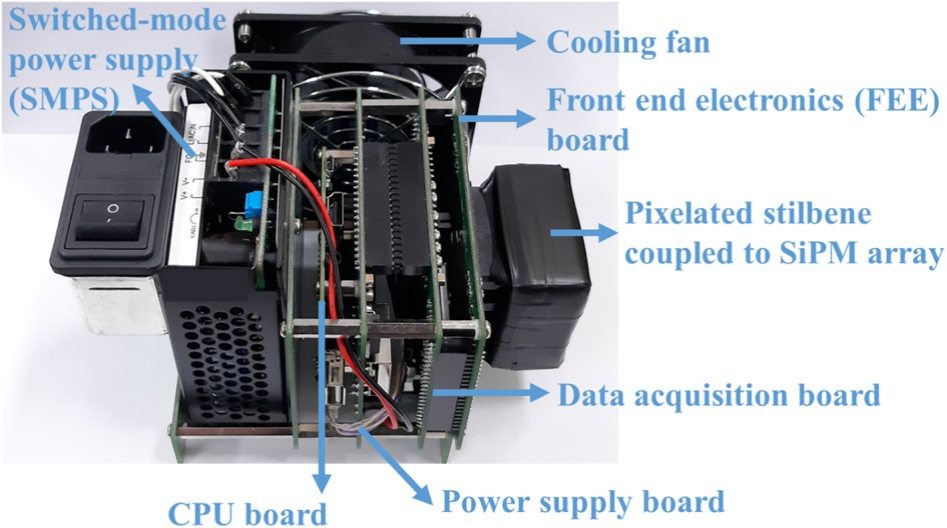
Hardware configuration for driving a SiPM-based pixelated stilbene array in which each of the electronic components is identified as well as the coupling to the stilbene and SiPM.

The event energy and position values are stored in a buffer and transmitted to the internal computer that is used for running the MLEM algorithm from which the source image is reconstructed. The image data is then transferred via Wi-Fi or transmission control protocol/internet protocol (TCP/IP) to a remote side PC such as a desktop or laptop. The axial fan (UF-92B23 produced by Fulltech) is also equipped to cool down the heat emission of the ADCs and maintain the performance of SiPM independently of temperature, as shown in Fig. 14. The description of the prototype hardware configuration in the figure is detailed in the Device Characterization section. The power drive unit uses an adapter power of 5 V DC/6 A as input power. It is designed to meet the current required for device driving and can be replaced by a battery in the near future; front end electronics and data acquisition boards consume 5 V/3A, and CPU board consumes 5 V/2A.

**Figure 15:**
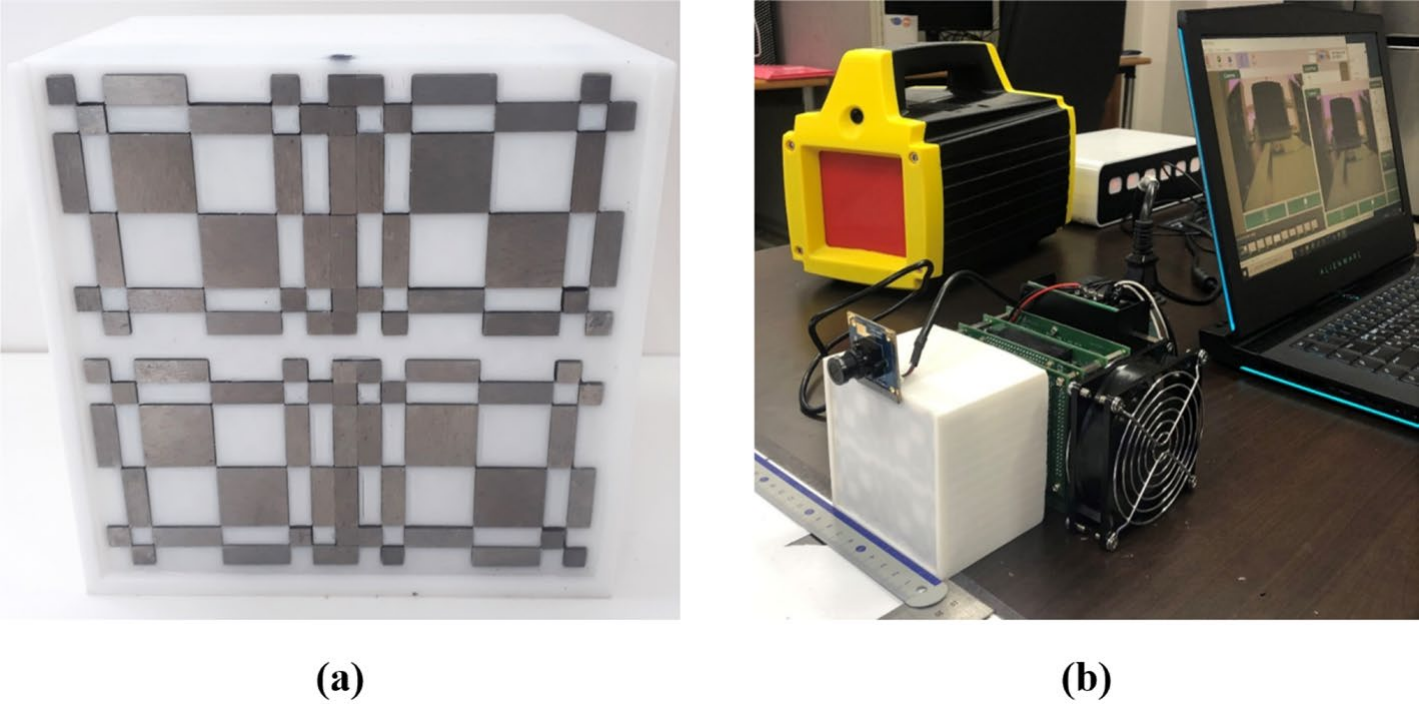
MURA mask (**a**), and developed hand-held DPI equipped with the MURA mask (**b**).

As shown in Fig. 15a, we developed a 21 × 21 rank, centered-mosaic, MURA mask composed of a 2 cm thick tungsten^12^. The tungsten-based mask was also chosen not only to block gamma-rays but also to scatter fast neutrons^26^. The mask selected an anti-symmetric quadratic residue array of size 21 × 21 (2p-1) with the prime number (p) of 11 to match the 11 × 11 pixel section of the SiPM. Previous work in our laboratory^3^ has shown that this MURA mask reduces measurement time by half and successfully mitigates the artifacts without using the conventional anti-mask method. Figure 15b shows the prototype hardware configuration equipped with the MURA mask. The total weight of the hardware, including the MURA mask and the tungsten shield (with a 1 cm thickness) enclosing the stilbene-SiPM array, is approximately 4.1 kg.

### Image reconstruction method

The image reconstruction method uses the MLEM algorithm as the iterative image reconstruction technique. The MLEM is based on the log-maximization of the Poisson-like probability function and described in the following equation^15^:

1 $${\lambda }_{j}^{new}=\frac{{\lambda }_{j}^{old}}{\sum_{i}{A}_{ij}}{\sum }_{i}{A}_{ij}\frac{{y}_{i}}{\sum_{k}{A}_{ik}{\lambda }_{k}+b}$$

where *y* contains the measured mask projection, in which *y*_*i*_ is the number of counts recorded by detector pixel *i.* That is, a point source is present in the projection because the projection of the source through the mask is deposited on the detector. *A* is the system matrix consisting of the estimated mask projections from various source positions, where *A*_ij_ is the predicted response of detector pixel *i* when the source is located in source-plane pixel *j*. If there is a mismatch between the measured and estimated projection (*λ*_k_, the ratio of two sets of projection), modifications are made to improve the estimate, and a new iteration is performed until *k*, the maximum iteration counter, is reached. This process leads to the maximized probability (λ_j_) when the source is located at a source plane pixel *j*. The term *b* is a noise term that represents the probability derived from the background radiation. Therefore, this process can readily estimate the position of the radiation source with a small number of incident radiation enough to form the mask projection. This procedure was implemented in MATLAB (MathWorks Inc, USA).

### Neutron/gamma separation using PSD

**Figure 16:**
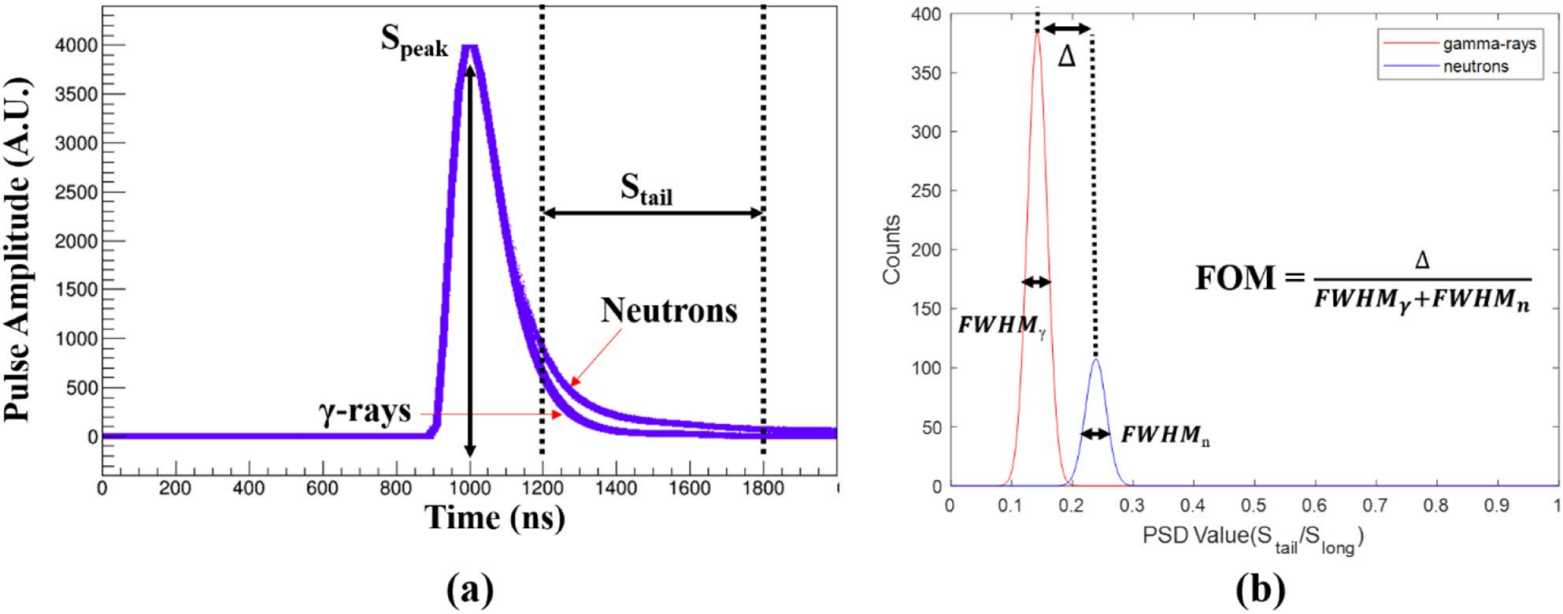
Illustration of the PSD method (**a**) and FOM evaluation (**b**) used in this study.

The charge comparison (CC) method was used in the PSD performance test, where the difference in the amount of delayed light produced by proton and electron recoil is used to identify neutron and γ-ray events, respectively. The capsule type ^252^Cf source which has a 3.145 MBq activity was used as a fast neutron and multi energy gamma-ray source. Because ^252^Cf emits an average of 3.759 neutrons and 8.3 gamma-rays per spontaneous fission event with a branching ratio of 3.09%^27^, the source emitted 3.5 × 10^5^ n/s into 4π and 7.8 × 10^5^ γ/s. The *PSD* value is given by the equation:

2 $$PSD= \frac{{Q}_{tail}}{{Q}_{peak}}= \frac{{\int }_{{tail}_{start}}^{{tail}_{end}}Qdt}{{Q}_{peak}}$$

where *tail*_start_ and *tail*_end_ are the beginning and end of the tail signal, respectively, used for the integration of charge generated by delayed light, as shown in Fig. 16a. *Q*_peak_ corresponds to the charge of the peak due to prompt light. The sampling rate of the ADS5281 ADCs was set to 50 MHz, for which the sampling interval is 20 ns. The beginning of the tail used for total charge integration was fixed at 10 samples following the maximum peak amplitude, and the end of tail was fixed at 40 samples from the pulse maximum. Figure 16b quantitatively describes the index of *PSD* performance as the FOM defined by the ratio of the peak center difference to the sum of the total width at half the maximum (FWHM) of the two peaks.

### Device characterization

Figure 14 shows the developed hardware system which consists of three developed printed circuit boards or PCBs that have the following functions. The front-end electronics (FEE) board conditions the analog inputs from the SiPM channels, the data acquisition (DAQ) board contains the digitization and front-end processing electronics, and the power supply board supplies the power to the instrument’s components. A commercially available central processing unit (CPU) board is equipped with an advanced RISC machine (ARM) CPU based on the Linux OS that utilizes a Raspberry PI board. The CPU board performs (1) the image processing using the MLEM reconstruction method and (2) superimposes the resulting radiological images upon a concurrent optical image. The image data is then transferred to a remote side PC such as a desktop or laptop. The amount of data that can be transferred is up to 10 Mbyte per second, which translates into a neutron/gamma-ray count rate of up to 1,000,000/s^28^. The FEE board has 24 channels for each orthogonal X- and Y-direction outputs. The FEE board also has a temperature sensor to correct the photoelectric conversion gain of the SiPM depending on the temperature.
