# Author Correction: Compact lightweight imager of both gamma rays and neutrons based on a pixelated stilbene scintillator coupled to a silicon photomultiplier array

**DOI:** 10.1038/s41598-021-94986-9

**Published:** 2021-07-28

**Authors:** Jihwan Boo, Mark D. Hammig, Manhee Jeong

**Affiliations:** 1 Nuclear and Energy Engineering, Jeju National University, Jeju, 63243 Republic of Korea; 2 Nuclear Engineering and Radiological Sciences, University of Michigan, Ann Arbor, 48109 USA

Correction to: *Scientific Reports* https://doi.org/10.1038/s41598-021-83530-4, published online 15 February 2021

The original version of this Article contained a repeated error where the unit “MeV” was incorrectly given as “meV” in the Introduction section.

The original Article has been corrected.

